# Pharmacotherapeutic intervention in impulsive preschool children: The need for a comprehensive therapeutic approach

**DOI:** 10.1186/1753-2000-5-11

**Published:** 2011-04-13

**Authors:** Christina Stadler, Margarete Bolten, Klaus Schmeck

**Affiliations:** 1 Department of Child and Adolescent Psychiatry, Psychiatric Clinics of the University Basel, Schaffhauserrheinweg 55, CH-4058 Basel, Germany; 2 Department of Child and Adolescent Psychiatry, Psychiatric Clinics of the University Basel, Schanzenstrasse 13, CH-4056 Basel, Germany

## Abstract

Impulsive and aggressive behaviour symptoms often are serious problems in children, even already at preschool age. Thus, effective treatment approaches are requested. In this comment pharmacotherapeutic treatment approaches, first of all risperidone, their limitations and alternative psychotherapeutic approaches are outlined.

## Limitations of phamacotherapeutic approaches in preschool age

Given the high prevalence and chronicity of oppositional defiant disorder (ODD) and conduct disorder (CD), their effective treatment is a major public health challenge. Psychopharmacotherapeutic approaches to disruptive behaviour disorders like ODD and CD comprise antipsychotics and mood stabilizers and, in ADHD, mostly psychostimulants. The number of children receiving second-generation antipsychotics is constantly rising and has doubled in the United States in a five year period from 2001 to 2005 [1]. However, the prevalence of psychotropic medication in young children is quite different between countries. In a US-MEDICAID sample of 11'700 children and adolescents 2,4% of children aged 0-3 and even 9,4% of children aged 4-5 became new users of second-generation antipsychotics between 2001 and 2005 [1]. In comparison, the prevalence of psychotropic medication in a German general population sample of 17'450 children was 0,18% in 0-2 year olds and 0,26% in 3-6 year olds (about one third of the medication were antipsychotics) [2].

Psychopharmacotherapy with risperidone appears effective in the first instance for reactive types of aggression as its effectiveness is mediated by a reduction of impulsivity, which is biologically determined to a certain extent [3]. The study conducted by Ercan and colleagues (2011, this issue) indicates that risperidone is effective also in preschool children with conduct disorder in reducing externalizing behaviour symptoms. However, side-effects of psychopharmacological treatment have to be considered especially in young children.

Correll et al. [4] studied the cardiometabolic risk of second-generation antipsychotics during first-time use in 505 children and adolescents aged 4-19 (22.1% suffered from disruptive/aggressive behaviour disorders). After 10 weeks of treatment with risperidone dyslipidemia developed in 19.4% and triglycerides increased significantly (p = 0.04). Weight gain ≥7% occurred in 64.4% of patients treated with risperidone (the only substance that showed higher rates of weight gain was olanzapine). Several studies have revealed that younger age predicts higher body weight gain under antipsychotic treatment [see for example [5]]. These results have to be taken seriously as there is a link between abnormal childhood weight or metabolic status and adverse cardiovascular outcomes in adults [6].

Beside these concerns we have to keep in mind that pharmacotherapeutic interventions are not effective beyond the treatment period. Despite its acute effect in reduction of impulsive outbursts, risperidone has not been shown to produce long-term changes in achievement or long-term prognosis. Therefore the use of second-generation antipsychotics like risperidone for use in children with disruptive behaviour disorders has to be discussed thoroughly and lower-risk alternatives have to be taken into account. Non-pharmacological approaches should play an important role in the treatment of ODD and CD aiming at reducing core problems of highly impulsive preschool children.

Regarding the multidimensional aetiological and mediating factors in the development of CD and ODD, comprehensive intervention approaches have to be considered in order to reduce not only acute symptoms, but also negative long-term effects. A treatment approach addressing only specific aggression mediating factors does not give sufficient consideration to the multiplicity of associated individual and environmental risk factors. Besides health factors, like birth complications or maternal smoking, especially psychosocial and parental factors have to be considered. A child's risk of developing ODD and CD is increased by parent psychopathology: Maternal depression, paternal alcoholism and/or criminality and antisocial behaviour in either parent [7,8] have been specifically linked to disruptive behaviour disorder. Since parental psychopathology like alcohol abuse or an antisocial personality disorder are among the most relevant risk factors for a persistent course of conduct disorder [9], an intervention has to target not only the children with behavioural problems but also their parents. This also implies families with high family burden like single parents, very young mothers or families with an adverse socio-economic status, but most importantly children exposed to deprivation or maltreatment.

## Main targets of intervention: The impact of early environmental conditions

It was repeatedly shown that an early adverse rearing environment is associated with altered functioning in the hypothalamus-pituitary-adrenal (HPA) axis - one of the core stress response systems. Weaver et al. [10] have shown that a repeated or longer period of low maternal care (low licking and grooming and reduced arched-back nursing) is associated with attenuated HPA axis activity, increased glucocorticoid response to subsequent stressors and fewer glucocorticoid receptors in the hippocampus. Most interestingly, it was additionally shown in several animal studies that changes in the mRNA expression are one of the consequences of adverse maternal care. Deviations in the epigenetic regulation of hippocampal glucocorticoid receptor expression as a consequence of early maltreatment was also shown in a first human study: The epigenetic effects in suicide victims who were abused in childhood compared to suicide victims with no history of childhood abuse and controls were similar to the effects observed in rats with mothers showing low maternal care like low grooming and licking behaviour [11].

Thus, chronic and sustained early adverse environmental conditions lead to neurobiological and molecular changes predisposing to emotional and behavioural changes (irritability, anxiety or aggression) which may lead to psychiatric disorders later on. On the other hand, there are results showing that parent-child relationships may play an important role in children's developing self-regulatory capacities [12]. A sensitive and responsive parenthood constitutes an external protective mechanism to regulate stress response and enhance effective emotion regulation processes in infants [13,14].There are promising results revealing that especially early intervention programs that aim to improve parental attachment and the ability to regulate stress in children are suitable to normalize neurobiological processes like cortisol response to social stress [15,16]. Thus, psychosocial risk factors might increase the risk for the development of CD on the one hand, but there is compelling evidence that a responsive attentive parenting style is protective and might even diminish a biological determined vulnerability. Kochanska and colleagues [17] for example revealed that a secure attachment relationship can serve as a protective factor in presence of risk conferred by a genotype: Among preschool children who carried the short variant of the serotonin transporter gene (5-HTTLPR) which is associated with a deficient serotonergic functioning and thus more impulsive-aggressive behaviour those who were insecurely attached developed poor impulse control capacities whereas those who were securely attached developed as good impulse control strategies as children with the non-risk allel.

## How family-focused interventions might work

The first three years in a child's development are exceptionally important in establishing later emotional, cognitive and social functioning, and parenting during this period has been identified as being one of the most important influences [18]. As it has to be assumed that the origin of persistent aggressive behavior is due to child risk factors like a different temperament as well as an adverse environment in which ineffective learning of emotion regulation plays a key role, only multi-psychosocial interventions show consistently sustainable effects [19-21]. Parenting that is provided in infancy and early childhood plays a crucial role in the infants evolving brain structures, and their impact on emotion regulation [22], and their developing security of attachment [23]. Insecure attachment has been shown to be related to behavioral problems [22]. The ability to empathize and to understand other people's thoughts and feelings is also related to the quality of the early parent-infant relationship, and it is recognized that deficits in these areas of functioning are associated with increased levels of violence and criminality [13]. A prospective longitudinal investigation on early mother-child interaction as a predictor of children's later self-control capabilities indicated that responsive, cognitively stimulating parent-toddler interactions in the 2nd year predicted later measures of cognitive non-impulsivity and ability to delay gratification [24].

There is increasing evidence that an important mechanism of change within interventions for children with aggressive or antisocial behavior may involve changes in parenting skill as a substantial predictor of child problem behavior outcome [25]. Positive proactive parenting (praise, encouragement and warmth) has been shown to be strongly associated with high child self-esteem and social and academic competence, and to be protective against later disruptive behaviour and substance misuse [26]. Parenting and family interaction variables have been shown to explain up to 30 to 40% of child antisocial behavior [27]. Parenting practices characterized by harsh and inconsistent discipline, little positive parental involvement with the child, and poor monitoring and supervision, however, have been shown to be associated with an increased risk for child antisocial behavior. There is also a significant body of research underpinned by the social and operant learning theory, addressing the relationship between early parenting practices and child's behavioral problems. The Social learning theory posits that children learn how to behave by imitating the behavior modeled by others in their environment and so if this behavior is reinforced, it is likely to be repeated [28,29]. Thus, training parents to model more social appropriate behavior and beneficial ways to regulate emotions may be very efficient. The operant learning theory underlines the environmental antecedents and consequences for human behavior. Therefore techniques of positive and negative reinforcement of child's behavior, i.e. praising and rewarding the desired behavior and ignoring or consequences for the child's negative behavior by parents are important components of early family focused interventions programs [30]. Cognitive components of family treatments focus on the dysfunctional thinking patterns in parents, that have been associated with conduct problems in their children [31,32]. Typical cognitive distortions are for example, globalized "Black-and-White-Thinking". Thus, one minor impediment or problem may trigger a cascade of negative automatic thought (e.g. " My child is bad" or "I am a bad parent"), that lead to feelings of distress, hopelessness, low self-esteem or learned helplessness [33]. Therefore, family-focused interventions aim parents to learn how to reframe dysfunctional cognitions or misattributions and to coach them in the use of problem-solving and anger management techniques [34].

These findings suggest that early parenting plays a key role to child emotional and behavioral functioning. Therefore early interventions designed to improve parent-infant interaction in particular, and parenting practices more generally, are essential in promoting childrens' adjustment and mental health. Thus, it can be assumed that every therapeutic intervention for infants at risk as early as possible is the most effective approach to prevent devastating effects of adverse early environmental conditions on neurobiological adaptive processes and the development emotional and behavioural problems.

## Family based Interventions for preschool-age children

Family based interventions for preschool-age children can be defined as an approach to treat children's behavior problems by training parents to change their child's behavior in the home setting. Interventions with individual families or groups of families of preschool children have been successfully applied in the clinic and home settings [35]. Such treatments aim to change parental behavior (e.g., less directive, controlling, and critical, and more positive) as well as child behavior (e.g., less physically and verbally aggressive, more compliant, and less destructive), and parents perceptions of the children's behavior. Recent reviews [35,36] present a number of parent training interventions that show a good effectiveness for improving conduct-problem behavior in preschool-age children: e.g. The Incredible Years by Webster-Stratton [30], Parent-Child Interaction Therapy [37], The Preschool Program by Schweinhart and Colleagues [38] and Triple P (Positive Parenting Program) by Sanders an Colleagues [39].

## What is needed in the treatment of children with severe ODD and CD

However, in clinical practice, therapy is often stopped and higher doses of medication are added when parent counselling or another kind of intervention is not efficient instead of intensifying behavioural interventions. It was shown, however, that an intensification of behavioural intervention has a large impact on treatment effectiveness independent of pharmacological intervention [40]. Due to the naturalistic life situation in these treatment camps, aggressive children can directly practise problem solving strategies since most of the highly impulsive-aggressive children know how they should behave in conflict situations, but they cannot show adequate behavior when physiological arousal is high and cognitive processes are affected. Thus, training emotion regulation in direct conflict situations seems effective to ensure greater generalization of therapeutic effects.

Following the idea of Pelham's summer treatment approach, also in Germany and Japan Intensive-Behavioural Treatment approaches have been developed comprising highly intensive child management and parent training with good intervention effects [41-43].

Also Multi-Systemic-Therapy (MST) is a multimodal intervention approach focusing on the individual, family, and extra-familial systems with promising long-lasting therapeutic effects also in chronic severely aggressive adolescents, but also in children from the age of 6 [MST-CAN, [44]]. Thus, multimodal intervention approaches should always be considered as first-line interventions before treating children with neuroleptics. However, in case of comorbid diagnoses like ADHD an adequate medication is recommended.

Comprehensive clinical settings in mother-child day-care or inpatient settings seem additionally promising since they assure a greater parental involvement and thus a better transfer to the familial setting [45].

A further point to mention is the fact that without a profound exploration of symptomatology and comorbidity therapy success always will be limited. ODD and CD comprise quite heterogeneous diagnostic groups and longitudinal studies show that only 50% of childhood onset CD show chronic patterns of aggressive behavior [46]. In a significant group of CD children externalizing behavior is not the core symptom. Instead, very often masked internalizing problems like separation anxiety, posttraumatic stress disorder or depression are associated with aggressive symptoms in young children [47]. Thus, it has to be strengthened that psychopharmacotherapy with risperidone should not be a first-line treatment in these patients presenting distinct comorbid symptoms.

## Conclusions

In summary, it can be concluded that several interventions are effective in enhancing emotion regulation and problem solving skills in highly impulsive and aggressive children. Parent management training, parent-child interaction therapy, cognitive-behavioural approaches, and other multimodal approaches are more effective than individual psychodynamic or traditional unfocused and open-ended psychotherapy approaches [48,49]. With regard to the high comorbidity with other externalizing and internalizing disorders as well with learning disabilities and associated academic failure, successful intervention also has to focus on comorbid symptoms. The treatment with atypical neuroleptics like risperidone should only be one strategy since effective interventions are multimodal and usually require a combination of several components of psychotherapeutic interventions, case management as well as pharmacological and educational intervention. Thus, the optimum method appears to be an integrated approach that considers both child and family within a variety of contexts throughout the developmental stages of the child and family's life. Due to the heterogeneity of disruptive behaviour disorders, future research should focus on the study of biological and psychosocial correlates of specific subtypes of aggressive behaviour with possibly different aetiology and specific treatment needs.

## Competing interests

The authors declare that they have no competing interests.

## Authors' contributions

All authors have equally contributed to the manuscript. All authors read and approved the final manuscript.
